# A polygenic risk score model for psoriasis based on the protein interactions of psoriasis susceptibility loci

**DOI:** 10.3389/fgene.2024.1451679

**Published:** 2024-11-06

**Authors:** Charalabos Antonatos, Fotios Koskeridis, Christiana M. Ralliou, Evangelos Evangelou, Katerina Grafanaki, Sophia Georgiou, Konstantinos K. Tsilidis, Ioanna Tzoulaki, Yiannis Vasilopoulos

**Affiliations:** ^1^ Laboratory of Genetics, Section of Genetics, Cell Biology and Development, Department of Biology, University of Patras, Patras, Greece; ^2^ Department of Hygiene and Epidemiology, University of Ioannina Medical School, Ioannina, Greece; ^3^ Department of Biomedical Research, Institute of Molecular Biology and Biotechnology, Foundation for Research and Technology‐Hellas, Ioannina, Greece; ^4^ Department of Epidemiology and Biostatistics, MRC Centre for Environment and Health, Imperial College London, London, United Kingdom; ^5^ Department of Dermatology-Venereology, School of Medicine, University of Patras, Patras, Greece; ^6^ Department of Biochemistry, School of Medicine, University of Patras, Patras, Greece; ^7^ Centre for Systems Biology, Biomedical Research Foundation, Academy of Athens, Athens, Greece

**Keywords:** psoriasis, polygenic risk score, protein-protein interaction, genome-wide association study, risk prediction

## Abstract

**Introduction:**

Polygenic Risk Scores (PRS) are an emerging tool for predicting an individual’s genetic risk to a complex trait. Several methods have been proposed to construct and calculate these scores. Here, we develop a biologically driven PRS using the UK BioBank cohort through validated protein interactions (PPI) and network construction for psoriasis, incorporating variants mapped to the interacting genes of 14 psoriasis susceptibility (PSORS) loci, as identified from previous genetic linkage studies.

**Methods:**

We constructed the PPI network via the implementation of two major meta-databases of protein interactions, and identified variants mapped to the identified PSORS-interacting genes. We selected only European unrelated participants including individuals with psoriasis and randomly selected healthy controls using an at least 1:4 ratio to maximize statistical power. We next compared our PPI-PRS model to (i) clinical risk models and (ii) conventional PRS calculations through *p*-value thresholding.

**Results:**

Our PPI-PRS model provides comparable results to both clinical risk models and conventional approaches, despite the incorporation of a limited number of variants which have not necessarily reached genome-wide significance (GWS). Exclusion of variants mapped to the *HLA-C* locus, an established risk locus for psoriasis resulted in highly similar associations compared to our primary model, indicating the contribution of the genetic variability mapped to non-GWS variants in PRS computations.

**Discussion:**

Our findings support the implementation of biologically driven approaches in PRS calculations in psoriasis, highlighting their potential clinical utility in risk assessment and treatment management.

## 1 Introduction

Psoriasis is a common, chronic, inflammatory cutaneous disease, displaying a varying prevalence amongst different geographical locations and ethnicities, ranging from less than 1% in Africa to 3% in western Europe (Parisi et al., 2020). The estimated heritability of the disease was estimated as high as 70% in a large Danish cohort (Lønnberg et al., 2013), further highlighting the substantial implication of the genetic variability in its pathogenesis. The *HLA-Cw6* allele, mapped in the major histocompatibility complex (MHC) at the 6p21 chromosome region, has been thoroughly validated in numerous studies as a susceptibility psoriasis locus, participating in the self-antigen presentation in T cells, including the nucleic acid/LL-37 complex and the melanocyte-secreted ADAMTS5 protein (Tsoi LC et al., 2017; Capon F, 2017).

Nevertheless, the *HLA-Cw6* allele exhibits a diverse worldwide frequency (Chen and Tsai, 2018). Particularly, white Europeans with psoriasis report increased *HLA-Cw6* positivity compared to Asians with psoriasis (Chen and Tsai, 2018), yet this disparity is not fully aligned with disease prevalence, thus suggesting additional risk loci that underlie the disease etiology. This polygenicity has been unveiled from two, different approaches that have been developed and implemented throughout the last decades, genetic linkage analyses and association studies. Specifically, a recent genome-wide association study (GWAS) on >19,000 cases of European ancestry have unraveled part of the genetic architecture of the disease, identifying 63 independent loci that account for more than 28% of the estimated heritability (Tsoi et al., 2017). Functional analysis of the significant loci revealed abundant inflammatory-related pathways, including T cell differentiation and pro-inflammatory cytokine secretion, such as the NF-κB cascade. Contrary to GWA studies, linkage analyses investigate possible risk loci transmissions within family members for a predefined, heritable trait (Ott et al., 2015). While their incompatibility in annotating the above modest-effect variants has limited their application in multifactorial traits, linkage studies have uncovered fifteen different genomic loci, known as psoriasis susceptibility loci 1–15 (PSORS1-15), that contribute to the pathogenesis of psoriasis, as derived from the Online Mendelian Inheritance In Man (OMIM^®^; http://www.ncbi.nlm.nih.gov/omim) (Amberger et al., 2009). Protein-coding genes mapped in the respective PSORS loci participate in both immune-related mechanisms, such as antigen-presentation and cytokine/chemokine signaling pathways, as well as mechanisms that govern the epidermal barrier and keratinocyte proliferation (Gunter et al., 2019). Interestingly, not all these loci have been identified from psoriasis GWAS with genome-wide significance, such as PSORS8 and PSORS9 (Capon, 2017).

Despite the considerable progress conducted in the identification of the genetic predisposition of psoriasis, little effort has been made towards the discrimination of individuals through their germline genetic risk. Stratification of individuals based on their genomic profile poses as an appealing strategy for enhancing the clinical practice in the context of preventive medicine and diagnosis. Polygenic risk scores (PRS) have been used extensively for risk stratification for several complex diseases including psoriasis. PRS use the genetic variation of an individual weighted by the effect size estimated from GWAS to assess its heritable risk of developing a specific complex disease (Lewis and Vassos, 2020). In psoriasis, studies covering PRSs calculations have, thus far, a minimal fraction of associated loci with SNPs selected based on a *p*-value threshold (Tsoi et al., 2017; Yin et al., 2015; Chen et al., 2011) excluding therefore an abundance of SNPs that might interplay a further, uncharacterized functional role in the disease etiology (Antonatos et al., 2023). However, aberrant interactions occurring between the encoded proteins (protein-protein interactions; PPIs) at a pathological state, perturbating the individual’s homeostasis, leads to the onset and progression of the disease, enabling the identification of the respective hub genes that are implicated in the pathogenesis of psoriasis (Kuzmanov and Emili, 2013).

Here, we evaluated the discriminative ability of a PRS model based on the PPIs of the PSORS loci, compared to rigorous statistical approaches through *p*-value threshold and a clinical risk model for psoriasis in the United Kingdom Biobank (UKB) cohort. We describe the process of constructing the PPI network, annotation of the corresponding genes and we (i) calculate the magnitude of strength of the association with psoriasis and (ii) its discriminative ability through receiver operating characteristic (ROC) curves.

## 2 Materials and methods

### 2.1 Study participants

We selected participants from United Kingdom Biobank, a population-based cohort study of over 500,000 individuals, aged 40–69 years old at baseline (Research Ethics Committee approval number: 21/NW/0157), who were recruited from across the United Kingdom and underwent an extensive genotypic and phenotypic characterization (Sudlow et al., 2015). Genotyping of the participants was performed using a custom Affymetrix array and imputations were performed centrally. In total, genetic data for 487,410 participants were available. Detailed information of the genotyping and imputation workflow is provided elsewhere (Bycroft et al., 2018).

One random participant from each pair of at least third-degree relatives (kinship coefficient>0.0884) as well as non-Europeans were excluded from further analyses. We used the inpatient Hospital Episode Statistics (HES) records to define psoriasis cases. All clinical psoriasis cases (L40.0-L40.9) were selected through the International Classification of Diseases, 10th Revision (ICD-10) medical coding system (Steindel, 2010). From the group of healthy participants, we randomly selected controls using an at least 1:4 ratio over cases to achieve maximum statistical power.

### 2.2 Protein-protein interactions of the PSORS loci

Protein-encoding genes, mapped to the PSORS loci, were identified through the OMIM database (https://www.ncbi.nlm.nih.gov/omim) (Amberger et al., 2009) and submitted to two continuously updated meta-databases of protein interactions; Protein Interaction Knowledge (PICKLE) 3.0 (Dimitrakopoulos et al., 2021) and InnateDB (Breuer et al., 2013) (Access date: 2 November 2021). PICKLE 3.0 includes both human and mouse experimentally determined PPIs and highlights such direct interactions via the reconstruction of the genetic ontology network, utilizing the reviewed human proteome of UniProtKB/Swiss-Prot as a reference panel (Apweiler et al., 2004), thus aiding to the clarification of the interactome implicated in the cutaneous inflammation. On the contrary, the predominant role of the innate immune response in the pathogenesis of psoriasis through the activation of dendritic cells and secretion of pro-inflammatory cytokines (Sweeney et al., 2011) was covered by the InnateDB, a manually curated meta-database which integrates interactions and pathway information participating in the innate immunity. To validate our exhaustive list of PSORS-interacting genes, we submitted all identified protein-encoding genes to the STRING v11.5 database (Szklarczyk et al., 2021). Considering the STRING v11.5 database, we utilized both functional and physical interactions between the submitted proteins, derived from seven different types of evidence used to predict the associations, with an interaction score at the default value of 0.4. Given the considerable variability in interactomes and specific interconnected modules, analyses conducted within such modules should be approached as exploratory.

Functional enrichment of the identified proteins from both databases was incorporated in our study as an additional validation method for the respective pathways, performed through over-representation analysis (ORA) using the Reactome Pathways database (Yu and He, 2016) with the R package clusterprofiler v.4.2.2 (Wu et al., 2021). *p*-values regarding the enrichment analysis were calculated with the hypergeometric test and controlled with the Benjamini and Hochberg false discovery rate (FDR) method; adjusted *p*-value ≤ 0.05 were considered as statistically significant.

### 2.3 Annotation of the PPI SNPs

Genomic locations of the PSORS-interacting genes were downloaded from the Ensembl database (Cunningham et al., 2022), including solely exonic and intronic regions to assess the discriminative ability of variants mapped to coding sequences. Genetic markers of the summary statistics were filtered for their chromosomal location, based on the genes under study for the PPI approach. Genome was split into 1703 non-overlapping approximately independent autosomal genomic loci as computed from the Berisa and Pickrell (2016) study using LDetect in the 1,000 Genomes Phase 1 dataset (Berisa and Pickrell, 2016), and widely used in further studies (Li et al., 2023).

### 2.4 Identification of highly interconnected modules

The interacting network from the 1,575, non-overlapping autosomal genes between both databases submitted into the STRING v11.5 database was visualized with the Cytoscape v3.9 software (Shannon et al., 2003). Highly interconnected clusters in the derived network were identified via the molecular complex detection (MCODE) algorithm using the default parameters (Bader and Hogue, 2003); We arbitrarily chose the top 6 identified clusters for additional PRS calculations.

### 2.5 Statistical analyses

All PRS in our study were constructed using a penalized regression (least absolute shrinkage and selection operator; LASSO) model, implemented in the lassosum v.0.4.5 R package (Mak et al., 2017), using weights from a published GWAS of psoriasis with 19,032 cases and 286,769 controls (Tsoi et al., 2017) from 8 different cohorts without UKB participants. Due to general access constraints, we used meta-analysis results without the 23andMe samples, including in total 13,229 cases and 21,543 controls. PRSs were adjusted for covariates including age, sex, first 4 genetic principal components and two major risk factors for psoriasis (Chalitsios et al., 2023), referring to smoking status (Data Field UKB code: 20116) and body mass index (BMI) (Data field UKB code: 21001).

Our primary analysis included comparisons of several models: (1) SNPs acquired from liberal *p*-value thresholding, including both relaxed and strict thresholds (*p*-value ≤ 0.1, 0.05, 0.005 and 5 × 10^−8^); (2) PPI-derived SNPs, where no *p*-value threshold was applied; (3) clinical risk model including age, sex, BMI and smoking status and (4) clinical risk model and PRS models. Secondary analyses encompassed (i) SNPs mapped to the top 6 highly interconnected modules identified in our gene list, (ii) SNPs mapped to the distinct protein-coding PSORS loci (1–9, 11–15) and (iii) two distinct analyses considering the *p*-value ≤ 0.1 and PPI-derived approaches without variants mapped in the PSORS1 locus.

Specifically, we calculated odds ratios (OR) and 95% confidence intervals (CIs) for each PRS model through logistic regression using standardized PRS values. The Wilcoxon signed-rank test was used to perform pairwise comparisons between the major PRS models. Discriminative ability of each PRS was assessed through the area under the curve (AUC) where we computed the c-statistic and the respective 95% CI with Delong’s method (DeLong et al., 1988), via 2000 stratified bootstrap replicates of the ROC curve through logistic regression. Briefly, the c-statistic provides the estimate that a randomly selected case has a higher PRS, or any other discriminative factor, than a randomly selected control. C-statistic values range from 0.five to one, with higher values indicating better classification. Ten-fold cross validation was also applied in our UKB dataset and c-statistics, accompanied by the respective 95% CIs. The pROC R package was used for the calculations of the c-statistic and corresponding 95% CIs (Robin et al., 2011). All statistical analyses were performed in the R software, version 4.1.2. A sample code for all analyses is provided at Supplementary File.

## 3 Results

### 3.1 PSORS-interacting proteins

**FIGURE 1:**
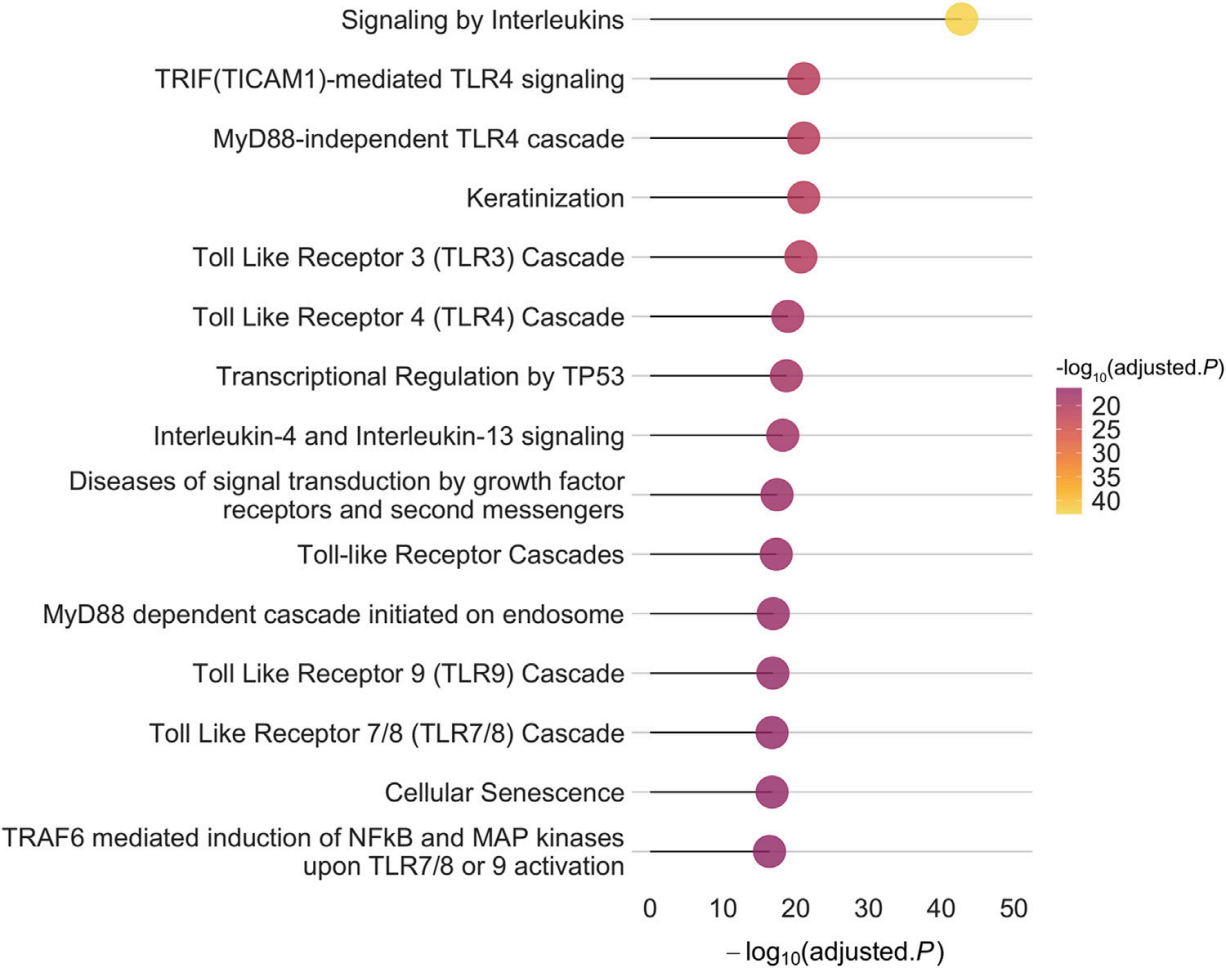
The top 15 enriched pathways based on the Reactome database for the 1,575 autosomal PSORS-interacting genes implemented in our approach. The *x*-axis represents the -log10 of the False Discovery Rate (FDR) adjusted *p*-value.

To construct the PPI-PRS, we identified 81 protein-coding PSORS genes mapped to 14 different chromosomal locations (Supplementary Table 1) through the OMIM database. We further investigated protein-protein interactions of PSORS genes using PICKLE 3.0 (Dimitrakopoulos et al., 2021) and InnateDB (Breuer et al., 2013), discovering a total of 1,373 and 1,045 interacting genes, respectively. More than half of the above genes were shared between both databases (728), with PICKLE exhibiting a larger divergence (645 genes) compared to InnateDB (317). Results from both meta-databases are presented in Supplementary Table 2. Overall, our systematic search revealed 1,575 non-overlapping PSORS-interacting proteins encoded by 1,575 autosomal genes (Supplementary Table 3). Genotyped autosomal SNPs, acquired from the largest GWAS on European psoriasis cases (Tsoi et al., 2017), were mapped to the PSORS-interacting gene list, corresponding to 360,710 independent variants; enrichment for the PSORS-interacting proteins, encoded by their respective genes, revealed 691 statistically significant enriched pathways (Supplementary Table 4), while the top 15 enriched Reactome pathways, according to their false discovery rate (FDR) adjusted *p*-value, are presented in Figure 1. Multiple toll-like receptor (TLR) and immune-related signaling pathways were significantly enriched in our gene list, such as signaling by interleukins and NF-κB activation, characterizing the inflammatory cascade observed during the psoriasis pathogenesis, while cellular, mitotic-associated processes including the transcriptional regulation by TP53, cellular senescence and keratinization further validated the aberrant hyperproliferation of keratinocytes in the cutaneous inflammation.

### 3.2 Comparison of PPI-PRS against conventional methods

**FIGURE 2:**
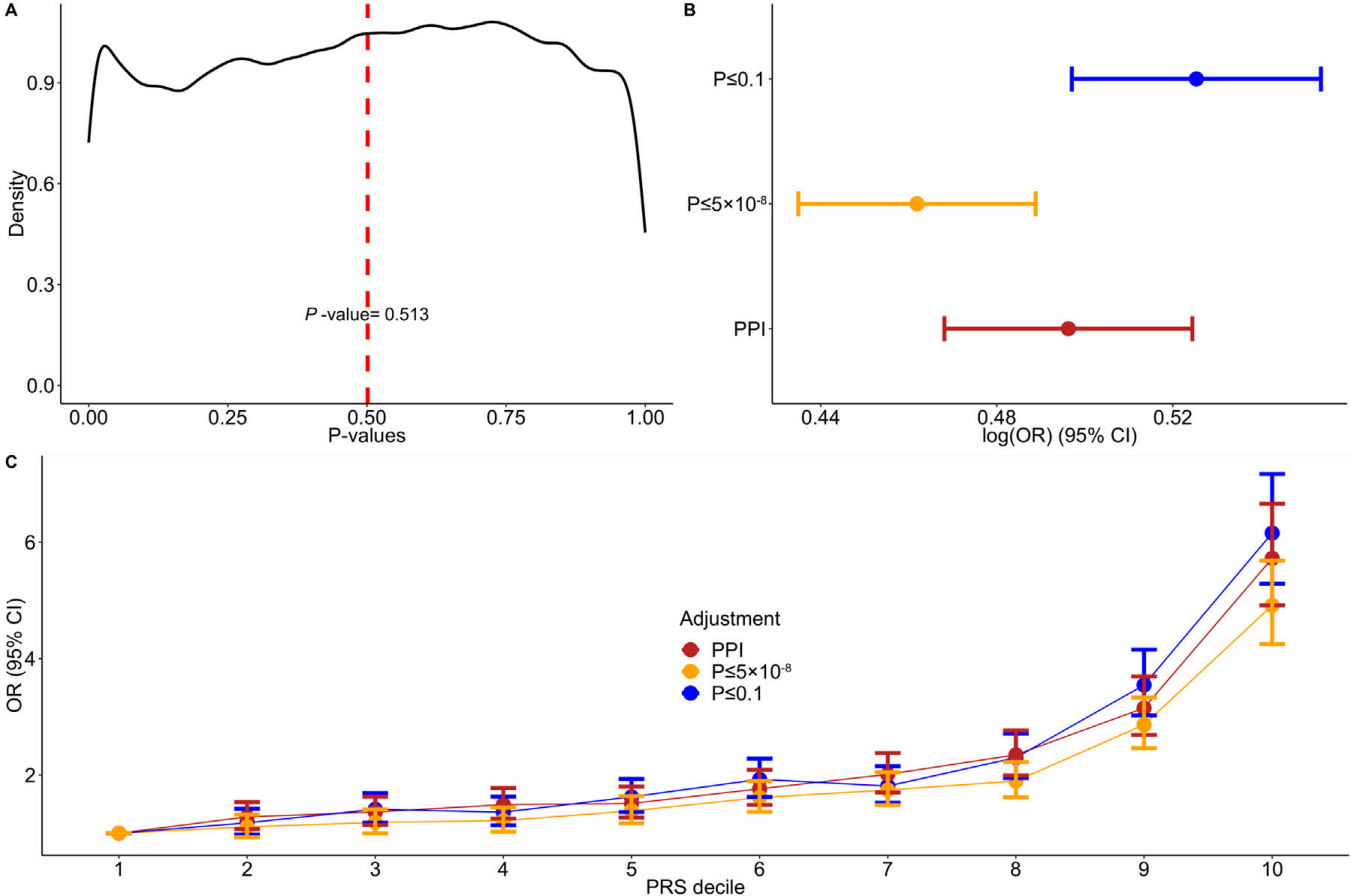
Associations between adjusted polygenic risk scores and psoriasis. **(A)** Kernel density plot of the variants’ *p*-values incorporated into the PPI model. **(B)** Forest plot showing the log (odds ratios) (log (OR)) and 95% confidence intervals (95% CIs) comparing the protein-protein interactions (PPI) model to *p*-value ≤ 0.1 and *p*-value ≤ 5 × 10^−8^ thresholding models. **(C)** Discriminative performance of our major polygenic risk score (PRS) models summarized according to OR. Estimated ORs and 95% CIs within each decile were estimated from logistic regression.

Our study consists of 4,434 psoriasis cases of European ancestry and 35,566 randomly selected unrelated controls from the UKB database. Descriptive characteristics are provided in Supplementary Table 5. Kernel density plot of the *p*-values of SNPs utilized in the PPI approach displayed various distribution peaks, with an average *p*-value of 0.5 (Figure 2A). Only 40,216 out of the 360,710 incorporated PPI SNPs (11%) had a significance level of less than 0.1, while 800/360,710 SNPs (4.2%) passed the threshold of *p*-value ≤ 5 × 10^−8^. We found that the PPI-PRS model is significantly associated with increased risk for psoriasis per 1-SD increase (log (OR) (95% CI) = 0.496 (0.468–0.524; *p*-value = 2.43 × 10^−261^; Supplementary Table 6), a result comparable with the conventional PRS approaches (overlapping 95% CIs) constructed by including SNPs with *p*-value ≤ 0.1 (log (OR) (95% CI) = 0.525 (0.497–0.553), *p*-value < 6.99 × 10^−290^) and *p*-value ≤ 5 × 10^−8^ (log (OR) (95% CI) = 0.461 (0.434–0.488), *p*-value = 2.9 × 10^−247^; Figure 2B). The Wilcoxon sign-ranked sum test indicated minimal, nevertheless significant differences between both *p*-value ≤ 0.1 (*p*-value = 0.001) and *p*-value ≤ 5 × 10^−8^ (*p*-value = 0.002) models. Increasing deciles of all major PRS approaches were also associated with a higher OR for psoriasis (Figure 2C).

**FIGURE 3:**
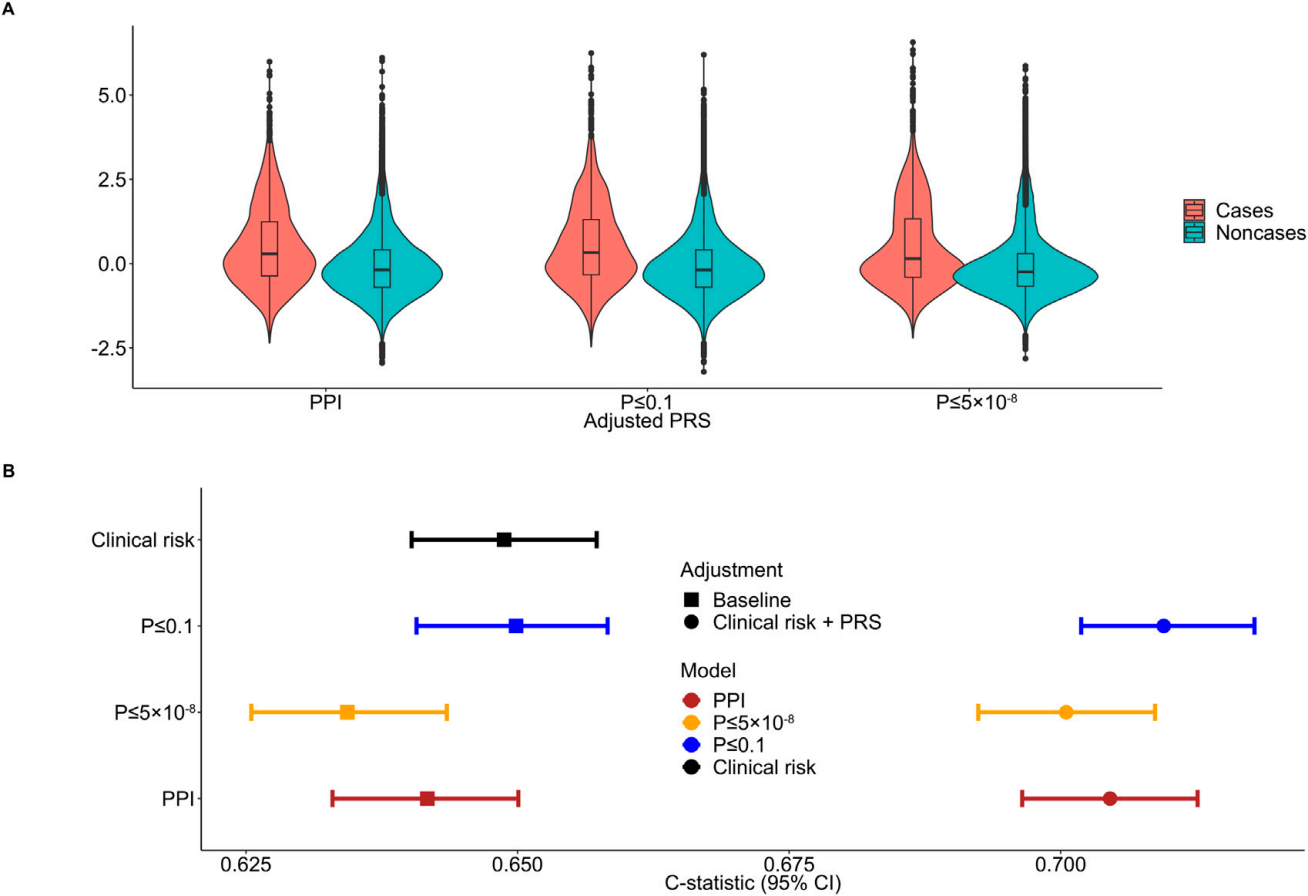
Distribution and discriminative ability of the clinical risk model, adjusted polygenic risk scores (PRSs) and the combined approach for psoriasis. **(A)** Standardized PRS distributions across the three primary models between psoriasis cases and noncases. **(B)** Forest plot showing the c-statistic and corresponding 95% confidence intervals (95% CIs) comparing the protein-protein interactions (PPI) model to conventional clinical risk model, *p*-value ≤ 0.1 and *p*-value ≤ 5 × 10^−8^ thresholding models and the combined approach. The clinical risk model includes age, sex, body mass index (BMI) and smoking status. Discrete color scales were used to discriminate between models. Discrete shape scales were used to discriminate between baseline and combined approaches.

All major PRS models showed overlapping distributions between psoriasis cases and non-cases (Figure 3A). The discriminative ability of the PPI-PRS model was estimated at 0.641 (95% CI: 0.632–0.650) as measured by the c-statistic (Figure 3B) (Table 1). This is almost identical with the c-statistic of the clinical risk model (c-statistic 95% CI): 0.648 (0.640–0.657); Δc-statistic (95%CI): 0.007 (−0.004-0.006)), *p*-value ≤ 0.1 threshold (c-statistic (95% CI): 0.649 (0.640–0.658); Δc-statistic (95% CI): 0.008 (0.001–0.015)) and *p*-value≤5 × 10^−8^ (c-statistic (95% CI): 0.634 (0.625–0.643); Δc-statistic (95%CI): 0.006 (−0.012-0.0002)) reporting overlapping 95% CI in all cases (Figure 3B). Ten-fold cross validation analyses across UKB dataset provided similar results (Supplementary Table 7). Stricter *p*-value thresholds, despite reducing the abundance of the genetic variants included, exhibited similar associations (Supplementary Tables 5, 6) and discriminative power (Supplementary Table 8) with overlapping 95% CIs compared to our primary PSORS-interacting approach.

**TABLE 1:** Number of Single Nucleotide Polymorphisms as well as c-statistic for the major approaches incorporated in our study. The reported Δc-statistic (95% CIs) corresponds to the PPI model.

Approach	Model	C-statistic (95% CI)	Number of SNPs	ΔC-statistic (95% CIs)
Baseline model	Clinical risk model	0.648 (0.640–0.657)	N.A.	0.007 (−0.004-0.006)
	*p*-value ≤ 0.1 threshold	0.649 (0.640–0.658)	1,015,916	0.008 (0.001–0.015)
	*p*-value ≤ 0.05 threshold	0.648 (0.640–0.657)	504,457	0.007 (−7.25 × 10^−6^-0.014)
	*p*-value ≤ 0.005 threshold	0.647 (0.638–0.656)	90,613	0.006 (−7.06 × 10^−4^-0.012)
	*p*-value ≤ 5 × 10^−8^ threshold	0.634 (0.625–0.643)	18,898	0.006 (−0.012-0.0002)
	PPI	0.641 (0.632–0.650)	360,710	N.A.
	Non-PSORS1 *p*-value ≤ 0.1 threshold	0.649 (0.640–0.658)	1,015,769	0.008 (7.79 × 10^−4^-0.015)
	Non-PSORS1 PPI	0.641 (0.633–0.650)	360,563	0.00 (0.00–0.00)
PRS + Clinical risk model	*p*-value ≤ 0.1 threshold	0.709 (0.701–0.717)	1,015,916	0.008 (−0.0001-0.016)
	*p*-value ≤ 0.05 threshold	0.709 (0.701–0.717)	504,457	0.007 (−0.0004-0.015)
	*p*-value ≤ 0.005 threshold	0.708 (0.700–0.716)	90,613	0.006 (−0.015-0.001)
	*p*-value ≤ 5 × 10^−8^ threshold	0.700 (0.692–0.708)	18,898	−0.006 (−0.015-0.001)
	PPI	0.704 (0.696–0.712)	360,710	N.A.
	Non-PSORS1 *p*-value ≤ 0.1 threshold	0.704 (0.701–0.717)	1,015,769	0.008 (2 × 10^−4^-0.015)
	Non-PSORS1 PPI	0.704 (0.697–0.712)	360,563	0.00 (0.00–0.00)

Abbreviations: SNP, single nucleotide polymorphism; CI, confidence intervals; PPI, Protein-Protein Interactions; N.A., not applicable; PSORS1, Psoriasis Susceptibility Locus 1.

When the clinical risk model was added to the PRS models, the 95% overlapping CIs were retained (Figure 3B). Notably, the Δc-statistic (95% CIs) of all combined models overlapped the null when compared to the PPI-PRS model (Table 1).

### 3.3 PRS of PSORS loci

To assess the association and the discriminative ability of the PSORS loci, we examined all variants mapped to the 81-protein encoding PSORS loci on our selected cohort. In total, 39,526 independent variants showed an association per 1-SD increase (log (OR) (95% CI): 0.427 (0.398–0.456); *p*-value = 3.45 × 10^−183^), generating a c-statistic of 0.615 (95% CI: 0.605–0.624). When considering each locus independently, PSORS1 (n_snps_ = 147) locus was associated with increased psoriasis risk (log (OR) (95% CI): 0.392 (0.364–0.420); *p*-value = 2.90 × 10^−164^), as well as the highest c-statistic with a marginal difference to the combined PSORS c-statistic (c-statistic (95% CI): 0.599 (95% CI: 0.590–0.608); Δc-statistic (95% CI): −0.015 (−0.018–0.011). Results from the association analyses, as well as ten-fold cross validation and c-statistics from the selected UKB patients for all independent PSORS loci are presented in Supplementary Tables 6, 7, 8.

### 3.4 Module-derived PRS

We further performed computations on highly interconnected modules present in our PPI network, visualized from the STRING v11.5 database as derived from the MCODE algorithm incorporated into the Cytoscape app. We arbitrarily explored the top 6 identified modules and performed PRS calculations on the incorporated genes. A gene list of all the highly interconnected modules identified from the MCODE algorithm is provided at Supplementary Table 9. Out of those, the fifth module encompassing the PSORS1 locus was associated with increased psoriasis (log (OR) (95% CI): 0.421 (0.393–0.450); *p*-value = 3.4 × 10^−187^), displaying the highest discriminative ability (c-statistic (95% CI): 0.613 (0.604–0.622) including 36,463 SNPs compared to all modules. The lowest discriminative ability was observed in the fourth module referring to keratin associated proteins, with a log (OR) of 0.052 (95% CI: 0.021–0.083; *p*-value = 8.7 × 10^−4^) and a c-statistic of 0.512 (95% CI: 0.503–0.521) encompassing 138 SNPs. All results are presented in Supplementary Tables 6, 7, 8.

The paramount contribution of the PSORS1 locus in the genetic predisposition of psoriasis, as displayed through both our module-derived PRS and our PSORS-PRS led us to the investigation of the discriminative accuracy of both major approaches excluding variants mapped in the above locus. The absence of the PSORS1 variants showed overlapping 95% CIs for both association and c-statistic computations compared to our primary approaches (Supplementary Tables 6, 7, S8), at both baseline risk models and when combining PRS and the clinical risk model (Table 1).

## 4 Discussion

In this study, we assessed a biological-driven approach as an additional way to shrink the abundance of genetic variants utilized in the calculation of PRS in the context of psoriasis, an inflammatory cutaneous disease with a divergent prevalence amongst geographical and genetic background (Parisi et al., 2020). PRS computational methods in psoriasis have, thus far, utilized a small fraction of the germline genetic variability *via* statistical thresholding (Antonatos et al., 2023). We hypothesized that the identification of experimentally validated protein interactions of the PSORS loci would allow us to study a wider spectrum of the disease pathogenesis, diminishing concurrently the genome-wide polymorphisms to pathogenesis-related ones. To evaluate the performance of our approach, we compared the magnitude of strength of the association and discriminative ability with conventional approaches at various *p*-value thresholds.

To fine-tune our model, we identified the protein interactions of the PSORS loci incorporating two meta-databases and explored the association and discriminative ability of our proposed SNP selection approach in the UKB cohort, using a total of 40,000 unrelated participants of European ancestry, including almost 4,500 psoriasis cases. We postulated that by identifying experimentally validated protein interactions of the PSORS loci, we would be able to investigate a more comprehensive spectrum of disease pathomechanisms while concurrently reducing remarkably the number of SNPs assessed. Indeed, functional enrichment of the 1,575 identified PSORS-interacting genes revealed multiple inflammatory pathways, mainly associated with antigen-presentation and cytokine signaling, as well as cell cycle pathways, representing the abnormal proliferation and differentiation of keratinocytes in psoriasis (Antonatos et al., 2023) (Figure 1). In addition, the independent SNPs mapped to the 1,575 autosomal PSORS-interacting genes evinced a heterogeneous *p*-value kernel distribution plot (Figure 2A); the PPI-PRS model composed largely overlapping 95% confidence intervals compared to both baseline clinical risk model and conventional *p*-value thresholding models (Supplementary Table 8). Strikingly, the Δc-statistic (95% CI) between the PPI-PRS model and *p*-value thresholding approaches overlapped the null in the combined approach (Table 1). Our above comparative analyses support the integration of prior biological insights during the selection of the germline genetic variants submitted to PRS models, thereby providing the framework for similar investigations in diseases with a complex genetic architecture, as well as application in cross-ancestry frameworks with available summary statistics and appropriate methodologies (Ruan et al., 2022).

We then sought to characterize the genetic risk of our PPI network through 6 highly interconnected modules and examine each PSORS locus independently. Despite the exploratory approaches conducted in the interconnected modules, we observed that PRS calculations regarding the PSORS1 locus (n_SNPs_ = 147), the protein-coding genes of all PSORS loci (n_SNPs_ = 39,562) and the 5^th^ module regarding our module-derived PRS computations -incorporating the PSORS1 locus- (n_SNPs_ = 36,463) showed nearly identical associations and discriminative ability, prompting us to investigate the implication of the PSORS1 locus in our primary PRS models. Notably, exclusion of the 147 PSORS1 SNPs resulted in highly similar associations and discriminative abilities with overlapping 95% CIs (Table 1), highlighting thus the contribution of the genetic variability mapped to non-GWS loci in the PRS computations.

Our study has a few caveats. In specific, our UKB cohort comprised of almost 4,500 individuals with psoriasis, disregarding for clinical subtypes with a possible distinct genetic background, fact that may affect the interactome utilized in our study. Assessment of such sub-phenotypes, for example, through the evaluation of biopsy specimens could further alter the discriminative ability of our proposed PRS model. Moreover, increasing research interest in protein interactions, leading to a pathological state, results in a continuous update of the meta-databases which could consequently alter our derived gene list. In addition, the protein interactome represents a substantial, nevertheless incomplete fraction of the deregulated mechanisms that lead to the onset of complex diseases. Implementation of the regulome, including noncoding RNAs (ncRNAs), could unveil genetic variants associated with the disease progression; SNPs mapped to such non-coding regions affect their gene expression and binding affinity, thereby contributing to the pathogenesis of psoriasis. Noncoding DNA regions are further referred to regulatory elements of a protein-coding gene, including enhancer/promoter regions, as shown in the largest genome-wide meta-analysis in psoriasis, where 13 loci were mapped to enhancers in cell types of the adaptive immune response (Tsoi et al., 2017; Antonatos et al., 2023). Additionally, GWASs have uncovered ample associated loci that map to genomic regions several kilobases away from protein-encoding genes, displaying their functional role via chromatin looping (Antonatos et al., 2023).

In summary, the development and application of a biological-driven PRS computation via the protein-interactions of the PSORS loci displayed a similar discriminative power compared to the rigorous, *p*-value thresholding approach. Both approaches, despite their relatively high predictive ability, are based on common variants provided by large-scale GWA studies. Our proposed pipeline provides biologically-driven insights into the genetic predisposition of psoriasis that could be further enhanced through the inclusion of rare variants and functional SNPs mapped to non-coding regions. Furthermore, the identification of PSORS-interacting genes linked to inflammatory and cell cycle pathways provides the framework for the discovery of novel therapeutic targets. By elucidating molecular mechanisms underlying psoriasis pathogenesis, these findings could guide future therapeutic interventions aimed at modulating these pathways, ultimately enhancing personalized treatment approaches for individuals with high genetic risk. Incorporation of such functional variants, combined with the heterogeneous environmental and clinical factors into a PRS model may aid the screening of individuals, especially those with a high germline genetic risk, and therefore precede the personalized risk prediction.

## Data Availability

The original contributions presented in the study are included in the article/supplementary material, further inquiries can be directed to the corresponding author.
