# Supplementary material for: A polygenic risk score model for psoriasis based on the protein interactions of psoriasis susceptibility loci
Source: Front Genet. 2024 Nov 6;15:1451679. doi: 10.3389/fgene.2024.1451679 (PMC11576467; doi:10.3389/fgene.2024.1451679)
Supplement: Supplementary file 1 [file Supplementaryfile1.docx]

Supplementary Material

Supplementary File. Sample code for all described analyses.

#####All scripts are based on GWAS summary statistics from Tsoi LC et al., 2017 and from UK Biobank (application number: 22102). Availability of GWAS summary statistics can be requested from the corresponding authors of the GWAS (doi: 10.1038/ncomms15382). Availability of UK Biobank individual-level data can be requested from the UK Biobank platform (<https://www.ukbiobank.ac.uk/>).

#####baseline c-statistic

baseline_glm <- glm(case~+age+sex+pc1+pc2+pc3+pc4+bmi+smoking, family=binomial(logit), data=data) #create predictors for baseline model

baseline_c_stat <- roc(baseline_glm$y, baseline_glm$fitted.values,

ci = TRUE,

boot.n=2000,

ci.alpha=0.95) #calculate c-statistic for baseline model

#####sample code for PRS calculation, association and ROC analyses

library(data.table)

library(lassosum)

library(pROC)

LDblocks <- "~/R/x86_64-pc-linux-gnu-library/4.1/lassosum/data/Berisa.EUR.hg19" # This will use LD regions as defined in Berisa and Pickrell (2015) for the European population and the hg19 genome

sum.stat <- "~/Workspace/sumstats" #GWAS summary statistics. In PPI and module-specific analyses, summary statistics were customized according to the mapped rsIDs in each genomic region of interest

bfile <- "~/Workspace/bfile" #bfile

covariate <- fread("~/Workspace/covariates") #a dataframe including age, sex, BMI, smoking status and 4 first genetic PCs

ld.file <- "EUR.hg19" #ancestry definition

prefix <- "Psoriasis"

target.pheno <- fread("~/Workspace/target.pheno")[,c("FID", "IID", "status")] #get FID, IID and case status (case/control)

ss <- fread(sum.stat)

cor <- p2cor(p = as.numeric(ss$p),

n = 40000,

sign = as.numeric(ss$beta)) #snp-wise correlations

fam <- fread(paste0(bfile, ".fam")) #read .fam file including all FID, IID

out <- lassosum.pipeline(

cor = cor,

chr = ss$chr,

pos = ss$pos,

A1 = ss$a0,

A2 = ss$a1,

ref.bfile = bfile,

test.bfile = bfile,

LDblocks = ld.file,

keep.ref = target.pheno[,1:2],

max.ref.bfile.n = 400000

) #lassosum pipeline

rsids <- ss[ss$pos %in% out$sumstats$pos, ]

write.table(out$sumstats$pos, "~/Workspace/SNPs", quote = FALSE, row.names = FALSE) #write file including all SNPs

v <- pseudovalidate(out, pheno = as.data.frame(target.pheno)) #pseudovalidation since no validation data were available

out2 <- subset(out , s = v$best.s, lambda = v$best.lambda) #get best lamda and s

res <- pseudovalidate(out2, test.bfile = bfile,

pheno = as.data.frame(target.pheno))

results <- test$results.table #get PRS

results$pheno <- replace(results$pheno, results$pheno == 0, "Controls") #replace 0 with controls

results$pheno <- replace(results$pheno, results$pheno == 1, "Cases") #replace 1 with cases

regr = glm(results$pheno ~ scale(results$best.pgs), family=binomial(logit)) #get unadjusted b and 95% CI for standardized PRS values

c_stat <-roc(results$pheno ~ scale(results$best.pgs),

ci = TRUE,

boot.n=2000,

ci.alpha=0.95) #get unadjusted c-statistic

table_prs = function(roc_analysis){

prs = data.frame(

FID = names(roc_analysis[["response"]]),

prs = roc_analysis[["predictor"]],

status = roc_analysis[["response"]]

)

prs[["FID"]] = gsub("_(\\d+)", "", prs[["FID"]])

prs[["FID"]] = as.integer(prs[["FID"]])

return(prs)

} #function to get a table including FID, standardized PRS and case status

table_all = table_prs(c_stat)

table_all = merge(table_all, covariate, by = "FID") #get a table with all clinical metadata and standardized PRS

regr_adj = glm(status~+age+sex+pc1+pc2+pc3+pc4+bmi+smoking+scale(prs), family=binomial(logit), data=table_all) #get adjusted b and 95% CI for age,sex, first 4 genetic PCs, BMI and smoking

rocs_covs_smoking[[model]] <- roc(regr_adj$y, regr_adj$fitted.values,

ci = TRUE,

boot.n=2000,

ci.alpha=0.95)

#####comparison of 2 PRS models

wilcox.test(PPI_PRS_table$prs, P_01_table$prs) #PPI_PRS_table contains standardized PRS values for the PSORS-PPI model, while P_01_table contains standardized PRS values for P<0.1 threshold

#####k-fold validation

all.response <- all.predictor <- aucs <- cis <- cis_psors <- c()

list_results_1 <- list()

Table <- data.frame(predictor = c_stat$original.predictor,

response = c_stat$original.response)

k <- 10

n <- dim(Table)[1]

indices <- sample(rep(1:k, ceiling(n/k))[1:n])

for (y in 1:k) {

test = Table[indices==y,]

learn = Table[indices!=y,]

model <- glm(response ~ predictor, data = learn)

model.pred <- predict(model, newdata=test)

cis <- c(cis, roc(test$response,

model.pred,

ci = TRUE,

boot.n=2000,

ci.alpha=0.95)$ci)

all.response <- c(all.response, test$response)

all.predictor <- c(all.predictor, model.pred)

}

list_results_1[[i]] <- cis #function that returns a list with c-statistic and corresponding 95% CIs for each k fold cross validation
